# Remission of erythromelalgia after semaglutide initiation: A case report

**DOI:** 10.1016/j.jdcr.2026.03.061

**Published:** 2026-04-09

**Authors:** Susanne Sofie Laugsand, Tore Wergeland, Sasha Gulati, Morten Engstrøm, Lars Erik Laugsand

**Affiliations:** a Department of Gastrointestinal Surgery, St. Olavs Hospital, Trondheim University Hospital, Trondheim, Norway; b Norwegian Centre for Headache Research (NorHEAD), Norwegian University of Science and Technology, Trondheim, Norway; c Department of Neuroscience and Movement Science, Norwegian University of, Science and Technology, Trondheim, Norway; d Department of Neurology and Clinical, Neurophysiology, St. Olavs Hospital, Trondheim, Norway; e Department of Neurosurgery, St. Olavs Hospital, Trondheim, Norway; f Department of Circulation and Medical Imaging, Norwegian University of Science and Technology, Trondheim, Norway; g Department of Emergency Medicine, St. Olavs Hospital, Trondheim University Hospital, Trondheim, Norway

**Keywords:** case report, erythromelalgia, GLP-1 receptor agonist, neurovascular pain, semaglutide, weight loss

## Introduction

Erythromelalgia (EM) is a rare neurovascular pain disorder characterized by episodic erythema, burning pain, and elevated skin temperature, typically triggered by heat or exertion.^1^ EM may occur in the context of autoimmune or prothrombotic conditions, or without identifiable cause.^1^ It remains challenging to manage, and pharmacologic treatments often provide incomplete or inconsistent relief.^1^ To our knowledge, there are no published reports describing complete and sustained EM remission following GLP-1 receptor agonist (GLP-1RA) therapy.

## Case report

A 46-year-old woman presented with a 4-year history of EM involving her hands and feet. Symptoms included nightly episodes of intense burning pain, erythema, and heat intolerance, relieved only by prolonged immersion in cold water. Outside of symptomatic flares, neurological examination was normal. No sweating abnormalities were reported.

Her grandmother had experienced similar symptoms, though never formally diagnosed with EM. Medical history included class 1 obesity (body mass index 33.9 kg/m^2^ at presentation), in addition to antiphospholipid syndrome (positive lupus anticoagulant), homozygous MTHFR C677T mutation, heterozygous prothrombin G20210A mutation, a prior transient ischemic attack, an episode of pulmonary embolism, and chronic migraine with aura. She had no history of diabetes, prediabetes, or known insulin resistance, and fasting glucose values had been consistently within the normal range. Her chronic medications included aspirin 75 mg daily for thrombosis prophylaxis and atenolol 50 mg daily for migraine prevention. She was not using topical agents, analgesics, neuropathic pain medications, over-the-counter drugs, or supplements at the time of presentation.

Laboratory evaluation, including complete blood count, inflammatory markers, thyroid function, and HbA1c (31 mmol/mol), was unremarkable. Autoimmune screening was negative, and no JAK2 mutation was found. Genetic testing of 24 genes, including SCN9A, showed no pathogenic variants. Skin biopsy from the right leg revealed normal intraepidermal nerve fiber density (12.5/mm; reference >5.7/mm). Nerve conduction studies were also normal.

Quantitative sensory testing (QST) at 5 sites showed mildly elevated cold and warm detection thresholds in a non–length-dependent and asymmetric pattern. Pain thresholds and dynamic allodynia were normal. Though isolated abnormalities were noted, the overall pattern was inconsistent with small fiber neuropathy and more in line with functional dysregulation.

The patient had previously trialed several treatment options. Increasing aspirin to 150 mg daily provided limited symptomatic relief. Misoprostol (0.2 mg twice daily) was discontinued due to abdominal cramps. She had not used gabapentin, pregabalin, or antidepressants due to concerns about side effects.

Semaglutide was prescribed for weight management due to obesity in the absence of diabetes, and was initiated at 0.25 mg weekly and titrated to 1.0 mg. By week 4, EM symptoms were resolved entirely. Her baseline body mass index at treatment initiation was 33.9 kg/m^2^, 33.2 kg/m^2^ at the time of symptom resolution after a 2 kg weight loss, and 23.2 kg/m^2^ after 15 months of treatment, corresponding to a total weight loss of approximately 30 kg. Semaglutide was initiated in January 2024 and has been continued at a stable dose of 1.0 mg weekly throughout follow-up, with no recurrence of symptoms. There were no medication changes aside from semaglutide. The only new symptom was mild cold intolerance in the hands and feet. Repeat QST showed normalization of thermal thresholds.

**Fig 1:**
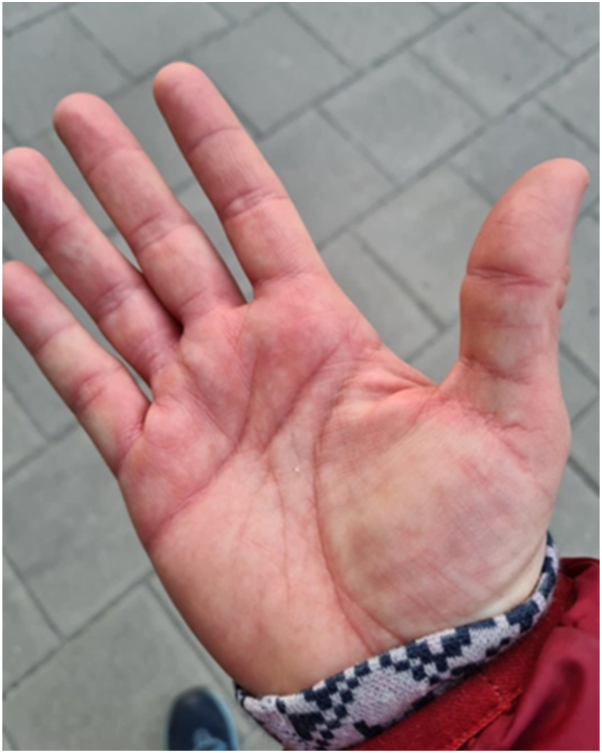
Erythema, swelling, and mottling of the hands during thermal flares before semaglutide therapy.

**Fig 2:**
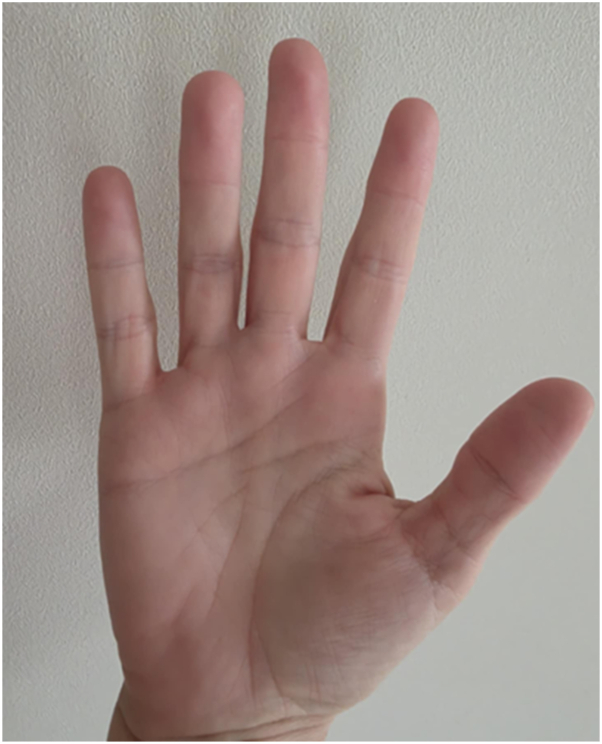
Normal appearance of the hands after semaglutide treatment, even in warm conditions.

Serial clinical photographs documented marked improvement. Before treatment, the patient exhibited distal erythema, swelling, and mottling (Fig 1). After semaglutide, her hands appeared completely normal under warm conditions (Fig 2).

## Discussion

EM pathogenesis involves complex interactions between microvascular and neurological dysfunction. Laser Doppler flowmetry studies have shown increased skin blood flow during flares, consistent with arteriovenous shunting and impaired nutritive perfusion. Histologic evidence supports microvascular instability.^2^^,^^3^

Some patients exhibit small-fiber dysfunction, although not all meet criteria for neuropathy.^4^^,^^5^ Normal intraepidermal nerve fiber density does not exclude erythromelalgia but argues against secondary small-fiber neuropathy. In this case, normal nerve fiber density and absence of thermal allodynia argued against small fiber neuropathy. The mild, non–length-dependent QST abnormalities may reflect reversible microvascular or autonomic dysregulation.

GLP-1RAs may modulate several pathophysiological pathways implicated in erythromelalgia. Anti-inflammatory effects include regulation of cytokine signaling and immune activation,^6^^,^^7^ while neuromodulatory actions involve suppression of microglial inflammasome activity and nociceptive signaling.^8^^,^^9^ Vascular effects include improvement of endothelial function and possible reduction of arteriovenous shunting, thereby enhancing nutritive capillary perfusion.^7^^,^^10^

Given the patient’s prothrombotic background, including antiphospholipid syndrome and inherited thrombophilia, a potential antithrombotic or microvascular effect of GLP-1RAs could be considered. However, the absence of laboratory evidence of active thrombosis and the rapid symptom resolution favor a mechanism related to modulation of vascular tone or autonomic regulation rather than inhibition of microthrombotic processes. Evidence regarding the effects of GLP-1 receptor agonists on other vasospastic disorders such as Raynaud’s phenomenon is currently limited, and no consistent clinical benefit has been established. The lack of evidence in this more prevalent vasospastic disorder underscores the need for cautious interpretation of mechanisms in erythromelalgia.

Reduced body mass may decrease heat retention and peripheral vascular load, potentially lowering the frequency of symptom triggers; however, remission occurred rapidly before substantial weight loss, arguing against this as the primary mechanism and suggesting a pharmacologic rather than purely metabolic effect. The development of cold intolerance may represent altered thermoregulatory setpoints or autonomic tone following treatment. The resolution of detection threshold abnormalities on QST supports a functional, reversible process.

To our knowledge, this is the first report of EM remission following GLP-1RA therapy. While causality cannot be established from a single case observation, the objective findings, including follow-up QST and photographs, support biological plausibility.

Limitations include retrospective review, absence of validated pain scoring, and lack of continuous temperature or perfusion monitoring. Additionally, responses to other GLP-1 receptor agonists may differ, and the observed effect cannot be generalized across the drug class. Nonetheless, this observation may generate hypotheses for future controlled studies evaluating GLP-1 receptor agonists in erythromelalgia and related neurovascular pain syndromes.

## Patient perspective

“For the first time in years, I could sleep through the night without pain. The cooling tubs I relied on became unnecessary. Regaining the ability to sleep and use my hands and feet normally has changed my daily life. I never imagined that a medication intended for weight loss could bring such dramatic relief from my symptoms.”

## Conclusion

This case illustrates a potential therapeutic effect of semaglutide in erythromelalgia. Given the growing use of GLP-1RAs and the unmet need for effective EM therapies, further investigation of their role in neurovascular pain syndromes is warranted. Reproducibility should be explored through structured clinical trials and mechanistic studies.

## Conflicts of interest

Dr Wergeland has received honoraria from TEVA, Roche, Lilly, Lundbeck, and holds stocks in Vilje Bionics AS and Keimon Medical AS. Drs Laugsand, Engstrøm, Gulati, and Author Laugsand have no conflicts of interest to declare.
